# Comparative Analysis of Laser and Plasma Surfacing by Nickel-Based Superalloy of Heat Resistant Steel

**DOI:** 10.3390/ma13102367

**Published:** 2020-05-21

**Authors:** Artur Czupryński, Bernard Wyględacz

**Affiliations:** Department of Welding Engineering, Faculty of Mechanical Engineering, Silesian University of Technology, Konarskiego 18A, 44-100 Gliwice, Poland; artur.czuprynski@polsl.pl

**Keywords:** nickel-based superalloy, heat resistant steel, surfacing, PPTA, HPDDL

## Abstract

In this article, the results of surfacing technology development, and structural, and mechanical properties examinations of 16Mo3 steel pipes with an outside coating of Inconel 625 deposited by automated plasma powder transferred arc (PPTA) and automated high power direct diode laser (HPDDL) surfacing were presented. Based on the results of non-destructive, metallographical macro- and microscopic, chemical composition, and thickness and hardness examinations optimal technology for use in high temperature energy or chemical industry applications was selected. The examinations conducted for each of the aforementioned technologies revealed the proper structure and high quality of coating. Dendritic structure with primary crystals growing in the direction of heat dissipation was revealed. No defects such as cracks, lack of fusion or porosity were found. Iron content in the most outer area of the layer made by PPTA with a heat input of 277–514 J/mm, thickness from 1.2 to 1.7 mm, between 4% and 5.5% was observed. Iron content in the most outer area of the layer made by HPDDL surfacing with output power of 1000–1600 W and scanning speed 3.3–4.7mm/s, from 0.6 to 1.3 mm in thickness, between 5.1% and 7.5% was observed. In coated pipes made by either technology high quality of surfaced layers, conforming to requirements posed on protective layers manufactured for prolonged exploitation in temperatures up to 625 °C, were observed. High temperature resistance examinations are the focus of further, yet unpublished, research. The obtained results point to slight differences in the parameters and properties of nickel-based superalloy layers surfaced on 16Mo3 pipes based on the technologies used. However, the process parameters optimization in the case of PPTA was simpler compared to HPDDL surfacing.

## 1. Introduction

The devolvement of the energy industry is determined by techno-economical, ecological, legal, but most of all material-technology factors [1,2,3,4,5,6]. Achieving energetic security and profitability is based on the correct design and application of proper high temperature resistant materials, the application of new and advanced manufacturing technologies, as well as the addition of capabilities for burning alternative fuels such as biomass, non-recyclable waste. In the manufacturing of machines and plants used in energy production, conversion, distribution, fuel processing, and storage high temperature and creep resistant steels are used, as well as more and more often other high temperature resistant metallic materials [2]. Parameters, operational period, and the need for periodic maintenance of machines and elements in the energy industry are based not only on technological and design parameters but mainly on the combined impact of temperature, external forces, mechanical and thermal cycling and chemical composition of the medium on metallic materials. 

The use of alternative fuels as an energy resource is a result of the international effort to reduce CO_2_ production. However, introducing organic matter and waste to fuel mix results in the production of highly reactive chlorides and fluorides, drastically increasing the rate of power plant elements corrosion [5,7]. The combined application of high temperature and corrosive environments induces a need for a change of used materials of application of protective layers in the power industry. Structural degradation of steel or layer, applied by means of surfacing or thermal spraying, on power industry elements operated in creep range is described by evolving with time set of structural and physicochemical properties depending on applied stress and exploitation temperature. Exploitation conditions of the designed element can lead to the formation and further development of gas pores, voids, and microcracks, leading to premature failure [4]. Structural instability of temperature resistant steels and nickel alloys is influenced by: level of substructural changes i.e., dislocation density evolution, recrystallization and recovery, intermetallic phase and carbide formation and dissolution, change of phase morphology (distribution, shape, size), decomposition of perlite, bainite and martensite, impoverishment of metal matrix in Cr, Mo, W. Aforementioned factors regardless of chromium content in steel or protective layer impact corrosion resistance including adhesion of passive layer, as well as, reduce material strength and resistance to cracking. The stability of this process is based on the chemical composition of the alloy, metal structure in the base state, and decides on the usage of high temperature steel or preventative surfaced/thermal sprayed layer in a given temperature, stress, and other environmental conditions [1,2]. The heat exchanger pipes in waste disposal plants are most susceptible to the corrosive environment. The fact results in a continuous decrease in parameters impacting the operational period and the occurrence of premature failures.

Pressure vessel steel 16Mo3 (1.5415) is a low alloyed chromium-molybdenum steel designed for application in elevated temperature, exhibiting high plasticity and ductility. Molybdenum, as a ferrite promoting element, has a high affinity to carbon. In pressure vessel steels with molybdenum content (e.g., 15Mo3, 16Mo3) there is a possibility of alloying elements carbides formation. Molybdenum content in steel reduces the temperature of perlitic transformation and has nearly no impact on bainitic transformation temperature. Molybdenum alloyed steels posses ferritic-perlitic, ferritic-bainitic, or bainitic microstructure [1,2]. Molybdenum addition in a range of 0.25–1% increases steel creep resistance, hardness, yield strength, and ultimate tensile strength, simultaneously decreasing elongation. Mo as an alloying element has a positive impact on brittle cracking resistance, impact toughness, decreases the critical cooling rate, and increases wear resistance. Moreover, Mo addition slightly decreases plastic workability and machinability. Chromium content in 16Mo3 steel increases corrosion resistance in steam environment. These grades of steels can be successfully applied in energy and chemical industries up to the working temperature of 530 °C (e.g., boilers, pressure vessels, hot medium pipelines) [4,5].

One of the methods used to increase the operational period of boiler elements in high temperature and corrosive environments is the usage of complex nickel-based alloy (superalloys) coatings. Nickel-based superalloys are characterized by very high resistance to the above-mentioned conditions compared to typical high temperature resistant steels. This metallic material group is characterized by the following properties: working temperature up to 1250 °C, limited susceptibility to cyclic and dynamic loading, resistance to nitrogen, sulfur, and carbon compounds [5,7,8,9,10]. A widespread group of nickel superalloys is Inconel. Inconel alloys were developed in the 1940s by a research group of Wiggin Alloy (Great Britan) and are still used in power, aero, and space industries among others [11,12,13,14,15,16].

Applied on the industrial scale mechanized, automated and robotized surfacing technologies still require complex studies, which lead to achieving high quality protective layers on pressure vessel elements posed by the evolving needs of the power industry. The most widespread fabrication method of Inconel-based protective layers is a consumable electrode in inert gas shielding surfacing—131 (metal inert gas—MIG) [2,10]. However, achieving high quality requires no surface or inner surfacing defects, low iron content in the surfaced layer, and the low heat affected zone (HAZ) depth is difficult or impossible in case of MIG surfacing. This fact induces the need for the application of modern and advanced coating technologies. The selection of proper surfacing, surfacing or thermal spraying technology for manufacturing nickel-based superalloy layers on pressure vessel steel is multivariable and requires extended studies [17,18,19,20,21,22,23,24,25,26,27,28,29,30,31,32,33,34,35,36,37,38,39,40].

For this reason, the present work is a study about the possibility of high power density methods application in manufacturing on Inconel 625 protective layers.

## 2. Experimental

### 2.1. The Aim of the Study

The aim of the study was to conduct a comparative analysis of basic technological parameters of automated PPTA surfacing and HPDDL surfacing on structure, chemical composition, geometry, base material content and mechanical parameters of nickel-based superalloy Inconel 625—NiCr 22M09Nb (2.4856) acc. EN 10095:2002 [41] surfaced layers on the outer surface of 16Mo3 (1.5415) acc. EN 10216–2 [42] heat resistant steel pipes.

Both assessed technologies were performed under a wide range of technological parameters to establish technical guidelines for producing surfaced layers conforming to the following criteria:

- Lack of surfacing defects,
- The thickness of a single surfaced layer in range 1–1.5 mm,
- Depth of HAZ ≤ 1.5 mm,
- Iron content in surfaced layer ≤ 7% [2,10],
- Relatively high process productivity in comparison with conventional surfacing methods.

The study covered among others:

- Determination of optimal process parameters range of surfacing based on preliminary technological trials,
- Assessment of geometry change and base material content in the surfaced layer depending on process parameters,
- Comparison of the microstructure of surfaced layer, heat affected zone and base material using metallography,
- Determination of atomic and weight composition of surfaced layers,
- Hardness testing of surfaced layer, heat affected zone and base material,
- Carrying and evaluation out of scratch testing.

### 2.2. Materials

The examinations were performed on a segment of 16Mo3 pressure vessel steel pipe 180 mm in length, 51 mm in outer diameter and the wall thickness of 5 mm surfaced with a singular layer of nickel-based superalloy Inconel 625 through robotized plasma transfer arc and laser surfacing. In both surfacing methods, additional material in the form of metallic powder 50–150 µm in diameter was used. The surfaced layer was not subjected to post surfacing heat treatment. Chemical composition of base and additional material, acc to the manufacturer and obtained by spectrometric analysis, are presented in Table 1, Table 2, Table 3 and Table 4. Mechanical properties of nickel-based superalloy weld metal are presented in Table 5. 

Prior to surfacing, according to the manufacturer recommendations, the powder was subjected to drying, by baking at a temperature of 200 °C for 10 min and mixed in a laboratory planetary stirrer. After preparation, the powder was placed in feeding hopper of a used powder surfacing station. The base material surface was prepared by cleaning the outer pipe surface from rust, mill scale, and grease. The base metal surface preparation consisted of abrasive blasting in cabinet sandblaster followed by mechanized brushing to remove any remaining electro corundum from surface and chemical degreasing. The used abrasive material was electro corundum normal brown with particle diameter 850–1000 µm (F22 acc. Federation of European Producers of Abrasives). The chemical agent used for degreasing was Tetrachloroethylene. Surface roughness parameters measured after preparation were Ra = 12 µm, Rz = 85 µm. The prepared pipes were mounted on an automated surfacing station. The station consisted of a horizontal pipe rotator (with self-centering chucks for pipe mounting) and pipe cooling equipment (with liquid coolant contact on the inner pipe surface). Additional station equipment changed based on surfacing technology applied.

### 2.3. Plasma Processing

PPTA was carried out with surfacing machine Durweld 300T PTA with machine powder plasma surfacing torch PT 300AUT (Durum Verschleiss-Schutz GmbH, Willich, Germany), mounted on industrial robot Fanuc R-2000iB (FANUC Ltd., Oshino-mura, Japan) arm. 

Based on a preliminary surfacing trial, nine parameter sets were used for the fabrication of samples. The initial evaluation of samples has shown the fabrication of surfaced layers of acceptable quality. Optimal parameters of automated PPTA enabling the formation of nickel-based superalloy Inconel 625 surfaced layers on Surface of pressure vessel steel 16Mo3 pipe of sufficient quality are presented in Table 6.

### 2.4. Laser Processing

The powder laser surfacing process was carried out on the robotized station, equipped with a modern laser system for surfacing mounted on a six-axis robot system, ABB IRB 2600 (Asea Brown Boveri, Zurich, Switzerland). A 2 kW 808 ± 5 nm wave length high power direct diode laser Rofin DL020 (ROFIN-SINAR Technologies Inc., Hamburg, Germany) with a rectangular beam with the top-hat intensity distribution in the slow-axis direction and a near Gaussian in the fast-axis direction was used in this study. The laser spot size in the focal plane, measured by the Prometec Laserscope UFF100, was approximately 1.8 mm × 6.8 mm. The fast-axis of the beam spot was set parallel to the traverse direction and the focal plane of the beam was positioned at the surface of the substrate material. The powder was injected directly into the molten pool by an off-axis powder injection system. To ensure a uniform powder distribution on the surface of the molten pool, the geometry of the powder injection nozzle has been fitted to the laser beam spot. Details of the used powder injection system are available elsewhere [12,16]. Argon was used as a shielding gas. In an effort to establish the range of optimal surfacing parameters, a series of single-pass clads have been made at laser powers of 1000, 1200, 1400, and 1600 W with traverse speeds ranging from 2.6 to 4.7 mm/s. The powder feed rate was in the range of 5 to 15 g/min. The optimal surfacing parameters (Table 7) were determined as the parameters providing the single-pass overlapping clad with a uniform and low fusion penetration into the base material.

**Figure 1:**
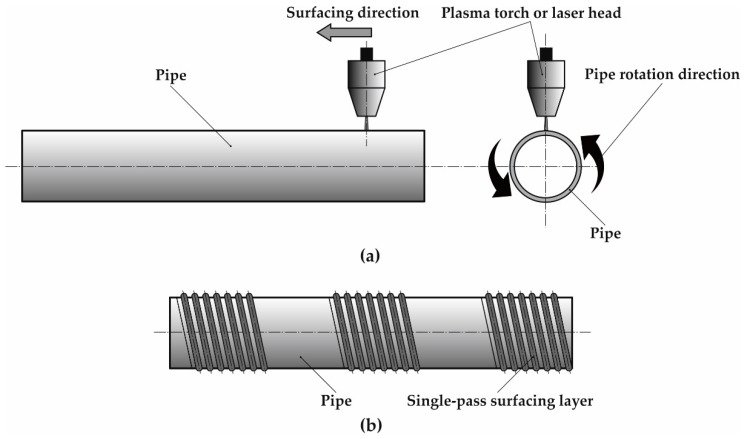
Schematic of outer 16Mo3 steel pipe surface surfacing with nickel-based superalloy Inconel 625: (**a**) movement of plasma/laser head and pipe rotation, (**b**) arrangement of surfacing passes on the pipe surface.

The schematic of outer 16Mo3 steel pipe surface surfacing with nickel-based superalloy Inconel 625 by powder plasma transferred arc surfacing and powder laser beam surfacing is presented in Figure 1.

### 2.5. Methodology of Research

To establish quality and reveal surfacing defects such as cracks, discontinuities, gas pores, non-uniform geometry or lack of surfaced layer adhesion visual (VT) and penetrant-dye (PT) testing were carried out on each sample. Surfaced layers properties evaluation was based on macro and microscopic metallographic examinations, surfaced layer thickness measurements, HAZ measurements, determination of base material content in surfaced layer, chemical composition analysis, and X-ray diffraction of the most outer area of surfaced layer, microhardness testing and surfaced layer scratch resistance testing. 

#### 2.5.1. Non-Destructive Testing

Visual and penetrant-dye testing was carried out in accordance with procedures, material, and equipment from normative ISO 17637 [44] and ISO 3452–2 [45]. Visual testing was based on a direct inspection with the naked eye of the surfaced layer surface procedure. Before the testing surface subjected to observation were cleaned and dried. Penetrant-dye inspection was carried out with the use of color contrast penetrant System Designation Type II, Sensitivity 2 (PT ISO 3452-2 II Cd-2). 

#### 2.5.2. Metallographic Examination

Microscopic examinations were carried out on a cross-section subjected to typical metallographic specimen preparation. The samples were etched in two stages: steel pipe base material structure was revealed by etching in FeCl3Et (Mi19Fe) solution, nickel-based superalloy structure was revealed by electrochemical etching in reagent: 20 cm^3^ HCl, 10g FeCl_3_, 30 cm^3^ distilled water. Etching parameters were selected by trial. Observation and recording of macro- and microstructure was carried out on Olympus SZX7 stereoscopic microscope (Olympus Corporation, Tokyo, Japan) and Olympus GX 71 inverted metallographic microscope (Olympus Corporation, Tokyo, Japan). Obtained macroscopic images enabled the determination of surfaced layer thickness, HAZ depth and base material content in the surfaced layer. Chemical composition examinations, including iron content determination, on the surface area of the nickel-based superalloy surfaced layer were done on XRF X-MET8000 Expert mobile spectrometer (Hitachi High-Technologies Corporation, Tokyo, Japan). Precise determination (surface and volumetric) of surfaced layer chemical composition were carried out on ZEISS SUPRA 25 scanning electron microscope (Carl Zeiss AG, Oberkochen, Germany) by means of Energy Dispersive Spectroscopy (EDS) method. High tension of 15 kV and probe current of 5 nA were used. X-ray diffraction testing, enabling phase content determination in surfaced layer, were carried out on X’Pert Pro PANalytical diffractometer (Malvern Panalytical Ltd., Malvern, UK) Cu (λ = 1.54056) lamp. X-ray diffraction was done in Bragg-Brentano geometry.

#### 2.5.3. Hardness Measurements

Surfaced layer hardness testing was done using Vickers method on Nexus 423D stationary hardness tester (Innovatest Europe BV, Maastricht, Nederland). Hardness testing was carried out acc. to ISO 6507 [46]. The test load used was 300 gf (2.942 N). Hardness tests were performed in 9 test points on polished metallographic transversal crosssection of chosen surfaced layers manufactured by powder plasma surfacing and powder laser surfacing. 

#### 2.5.4. Scratch Test

**Figure 2:**
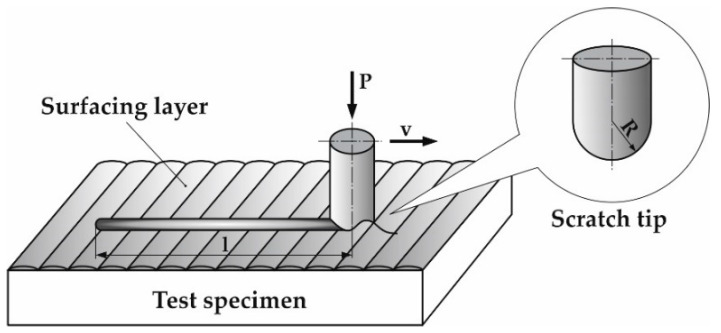
Schematic diagram of Inconel 625 nickel-base superalloy surfaced layer scratch test.

To establish scratch resistance and kinetic friction coefficient of surfaced layers, macroscale scratch tests—Revetest (RST) on Revetest Xpress Scratch Tester machine (Anton Paar Instruments, Graz, Austria) were performed. Scratch tests were carried out with the use of Rockwell diamond indenter of radius R = 200 μm. Constant Load Scratch Test (CLST) mode of scratch testing was used. The indenter travel direction was parallel to the pipe axis and velocity was v = 0.3 mm/min on test length l = 5 mm with constant load P = 100 N. Schematic diagram of the scratch test is presented in Figure 2.

## 3. Results and Discussion

### 3.1. Non-Destructive Testing Results

**Figure 3:**
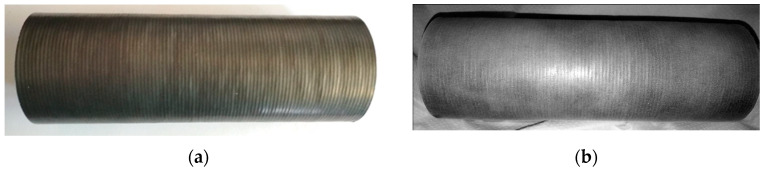
Photograph of Inconel 625 nickel-based superalloy surfaced layer on 16Mo3 steel pipe: (**a**) after surfacing, (**b**) after dye-penetrant application, removal and developer application (visible pipe length *L* = 180 mm, diameter *D* = 51 mm and thickness *t* = 5 mm).

During surfaced layers manufactured by powder plasma surfacing and powder laser surfacing no surfacing defects of type: cracks (100), surface pores (2017), excessive weld metal (502), incorrect weld toe (505), spatter (602) and other were found. Manufactured surfaced layers had high surface smoothness and symmetry of surfaced seam overlaps (Figure 3). Both powder plasma surfacing and powder laser surfacing enabled the formation of surfaced layers with quality level B. According to ISO 5817 [47] norm level B corresponds to the highest quality of manufactured layers.

### 3.2. Metallographic Test Results

**Figure 4:**
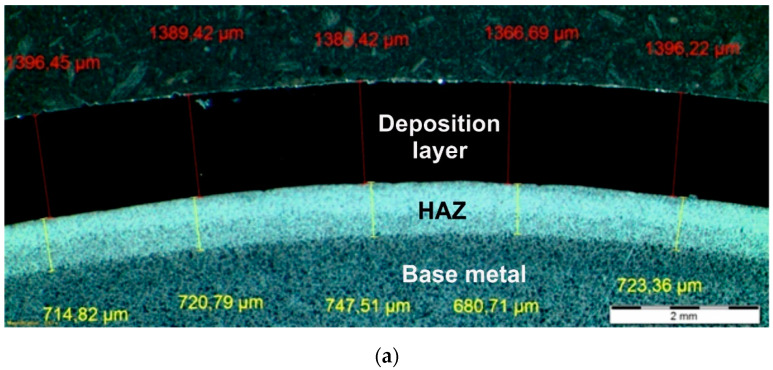

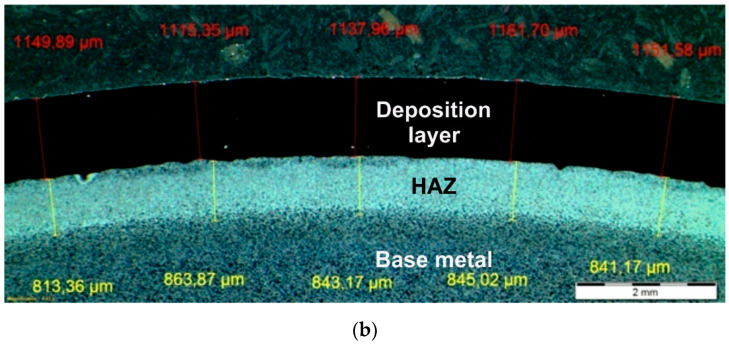
Sample macrostructures of the fusion line between nickel-based superalloy surfaced layer and steel pipe base material (**a**) powder plasma surfacing (sample P2), (**b**) powder laser surfacing (sample L4).

Carried out macroscopic examinations of Inconel 625 surfaced layer and 16Mo3 steel pipe base material did not reveal any defect such as: cracks, lack of penetration, gas pores, and other types of discontinuities in fusion line area both powder plasma surfacing and powder laser surfacing. Lack of defects presence in the fusion line area indicates correct parameter selection and sufficient preparation of the pipe surface. Sample macrostructures of the fusion line between nickel-based superalloy surfaced layer and steel pipe base material are shown in Figure 4. The measured thickness of nickel-based superalloy layer manufactured by powder plasma surfacing was 1.2–1.7 mm (Table 8). The measured thickness of nickel-based superalloy layer manufactured by powder plasma surfacing was 0.6–1.3 mm (Table 9). The measured values are lower than recommended 2.5 mm [8,9]; however, taking into consideration the high price of nickel alloy additional material and the difference in heat transfer coefficient between nickel- and iron-based alloys surfaced layers can be considered to be conforming to criteria for exploitation and optimal [35,36]. Surfaced layers were manufactured as a single layer surfaced without post-weld heat treatment.

**Figure 5:**
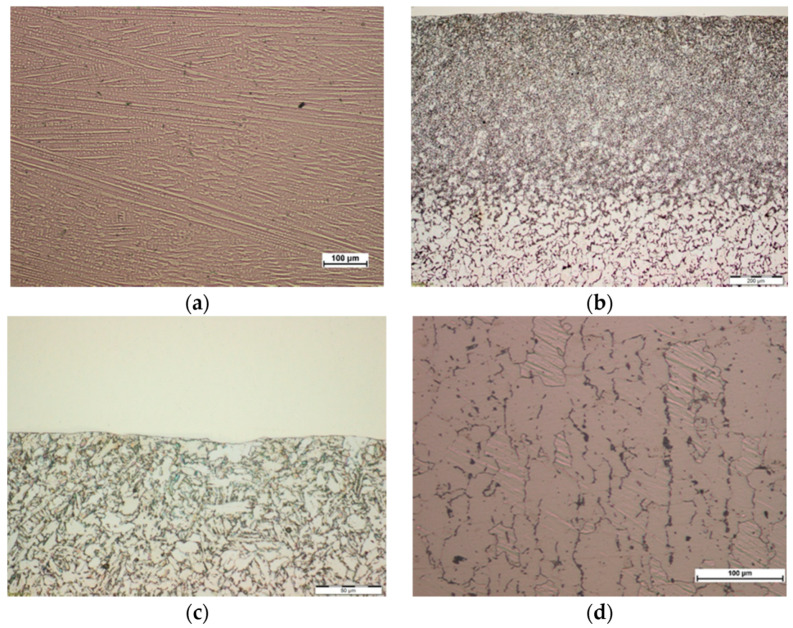
View of the microstructure of Inconel 625 superalloy coating layer obtained in the process of plasma powder transferred arc surfacing on the outer surface of 16Mo3 steel pipe (sample P2): (**a**) coating layer area, mag. 100×, (**b**) general view HAZ area mag. 100×, (**c**) HAZ area at the fusion line, mag. 500×, (**d**) base material, mag. 200×.

**Figure 6:**
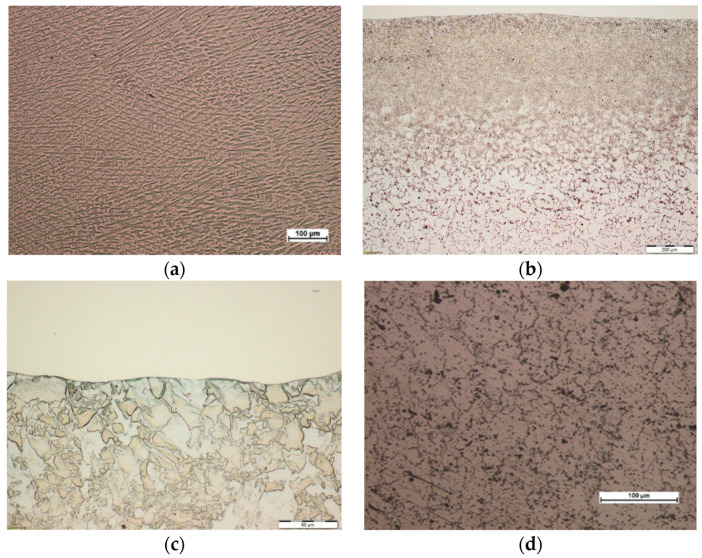
View of the microstructure of Inconel 625 superalloy coating layer obtained in the process of powder laser surfacing on the outer surface of 16Mo3 steel pipe (sample L4): (**a**) coating layer area, mag. 100×, (**b**) general view HAZ area, mag. 100×, (**c**) HAZ area at the fusion line, mag. 500×, (**d**) base material, mag. 200×.

Microscopic metallographic examinations revealed the ferritic-perlitic microstructure of 16Mo3 steel base material (Figure 5d and Figure 6d). In the case of both surfacing technologies in HAZ diversified microstructure was observed from martensitic, through martensitic-bainitic to ferritic-bainitic. Moreover, high heating and cooling rates of the base material in the heat affected zone, due to higher achievable surfacing velocity, have lowered grain growth rates observed in the case of powder plasma surfacing (Figure 5c and Figure 6c) [37,38,39]. In both powder plasma surfacing and powder laser surfacing depth of HAZ was low, a probable cause was liquid cooling of pipe inner surface during the process. Visible HAZ depth evolved with the change of heat input into the material. With the increase of surfacing linear energy depth of the base material layer subdued to microstructural changes. In the range of examined parameters slightly higher depth of HAZ occurred as a result of powder laser surfacing, but did not exceed 1350 µm (Table 9).

**Figure 7:**
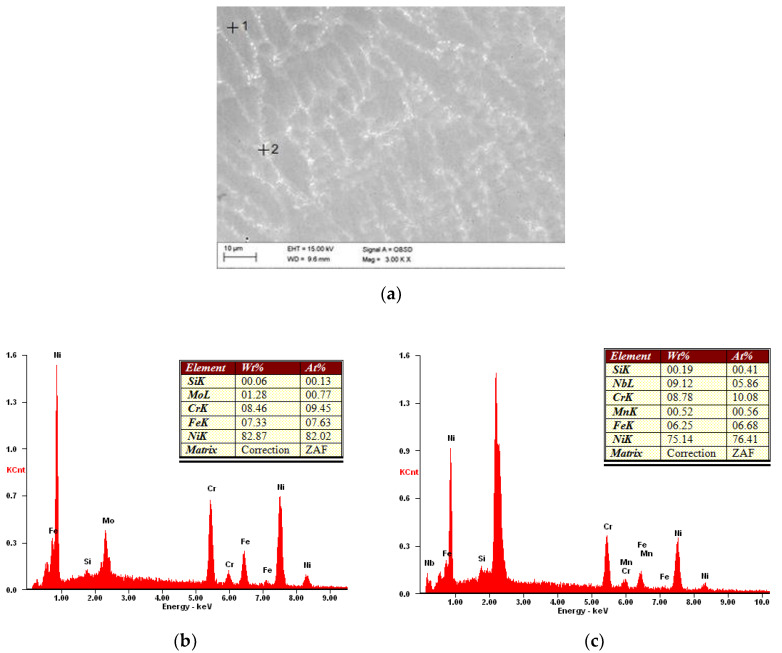
Sample BSE image of surfaced layer microstructure with results of EDS point microanalysis, mag. 3000×, high tension 15 kV (sample L4): (**a**) a view of dendritic structure, (**b**) chemical analysis of intradendritic area (measurement point 1), (**c**) chemical analysis of interdendritic area (measurement point 2), higher content of Nb compared to Inconel 625 powder chemical composition is notable.

Surfaced layers in each case were composed of organized, primary, fine-grained microstructure of dendrites and precipitations in interdendritic space—which is typical for nickel-based superalloy Inconel 625. Packets were orthogonal, dendritic, and oriented in the heat dissipation direction (Figure 5a and Figure 6a). The structure was highly uniform and possessed no gas pores or cracks on the microscopic level. Slight grain refinement on the boundary layer between surfaced material and base material was observed. (Figure 5b and Figure 6b). For each layer, slight fusion into the base material (enabling monolithic bonding of the surfaced layer with the pipe outer surface) was observed. One of the base criteria for the outer surface layer of heat exchangers pipe exploitation in biomass and waste fueled furnaces is the assessment of iron content in the surface layer. The guidelines of 7% maximal wg. content of Iron in case of automatic surfacing and 10% maximal wg. content of Iron in case of manual surfacing should not be exceeded [2,10]. Exciding the maximal iron content in the surfaced surface layer cand lead to iron oxide (Fe_2_O_3_) formation, which due to discontinuous and lamellar structure are prone to flaking during exploitation [2,10]. Measured iron content in the case of powder plasma surfaced layers, with the exclusion of sample P1, was in the range of 4.0–5.5 wg.% (Table 8), whereas in the case of powder laser surfaced layer only samples L4, L5, L6 had around 7 wg.% of iron (Table 9). EDS chemical composition microanalysis (Figure 7) revealed that the interdendritic area is rich in Niobium content. Iron and Chromium are equally present intra- and interdendritically. Iron presence in the surface layer is due to the mixing of base and additional metal. Chemical composition of interdendritic precipitations suggests the presence of γ/NbC eutectics, probably derivative of Ni2Nb Laves phase.

One of the main factors determining iron content in the surfaced layer is surfacing linear heat input. According to [7,11,15] the linear heat input should no exceed 300 J/mm. In the case of PPTA with intensive liquid cooling of the inner pipe surface, modified heat dissipation mode enabled obtaining of sufficiently low iron content in the surfaced layer when linear surfacing energy was kept under 500 J/mm. Maximum linear heat input into the material in case of powder laser surfacing, with other technology parameters unchanged, which resulted in the sufficiently low iron content was 340 J/mm.

### 3.3. Results of the XRD Analysis

**Figure 8:**
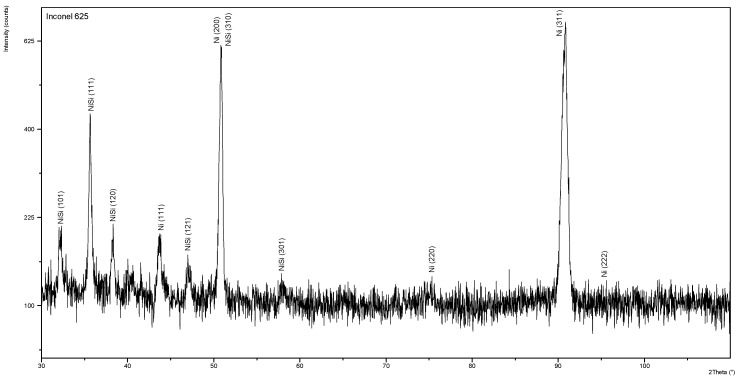
Examples of X-ray spectrum of overlay weld in the surface of Inconel 625 superalloy layer obtained in the process of plasma powder transferred arc surfacing (sample P2).

X-ray diffractogram for singular surfaced layer of nickel-based superalloy Inconel 625 manufactured by powder plasma surfacing (sample P2) was presented in Figure 8. Similarly in powder laser surfaced layer and powder plasm surfaced layers examinations main counts peaks from nickel are observed on diffractogram with angles: 2θ = 43.66°, 50.85°, 75.34°, 90.83°, and 96.74°. However, main count peaks for pure nickel crystalline structure from JCPDS-ICDD database angles are: 2θ = 44.51°, 51.85°, 76.37°, 92.94°, and 98.45°. In the case of analyzed sample counts peaks from crystallographic planes γ (111), γ (200), γ (220), and γ (311) were observed with slightly higher angle 2θ. This difference can be caused by the change of lattice parameters due to the solid solution of Inconel 625 alloying elements in Ni lattice and strengthening phases precipitations. In the surfaced layer Ni-Si phase with lattice Miller indices (101), (111), (120), (121), (301), and (310) was found. Moreover, in the surfaced layers manufactured by powder plasma surfacing or powder laser surfacing no other phases with parameters comparable to pure Ni were found.

### 3.4. Hardness Measurements Test Results

Measured in the course of macroscopic metallographic examinations of powder plasma surfaced pipes and powder laser surfaces pipes, the depth of HAZ (Figure 5) was confirmed in microhardness tests. Mean microhardness HV0.3 results tested on cross-section on 16Mo3 steel plates surfaced with nickel-base superalloy Inconel 625 manufactured by surfacing were presented in Table 10 and Table 11. The presented hardness results are mean from five measurements done at 0.1 mm intervals in the base material, HAZ, and surfaced layer. The base material was characterized by hardness in range 160–168 HV0.3. In the range of used surfacing parameters, the surfacing thermal cycle increased the hardness of HAZ to maximally 210 HV0.3 in case of plasma surfacing and 260 HV0.3 in case of laser surfacing. The HAZ microstructure evolved with the increasing distance from the fusion line. Near the fusion line bainitic-ferritic microstructure with a hardness of around 200–210 HV0.3, as a result of normalization, was present. In the distance of around 1.5 mm from the fusion line, microstructure changed to ferritic-pelitic-bainitic with hardness in the range 170–180 HV0.3. In the distance of 2.0 mm and more from the fusion line, the perlitic-ferritic microstructure of base material was present. The surfaced layer manufactured by powder plasma surfacing was characterized by hardness of over 240 HV0.3, while the hardness of the powder laser surfaced layer decreased sharply, from 261 HV0.3 to 187 HV0.3, with the increase of base material content in the surfaced layer (Table 11).

### 3.5. Scratch Test Results

**Figure 9:**
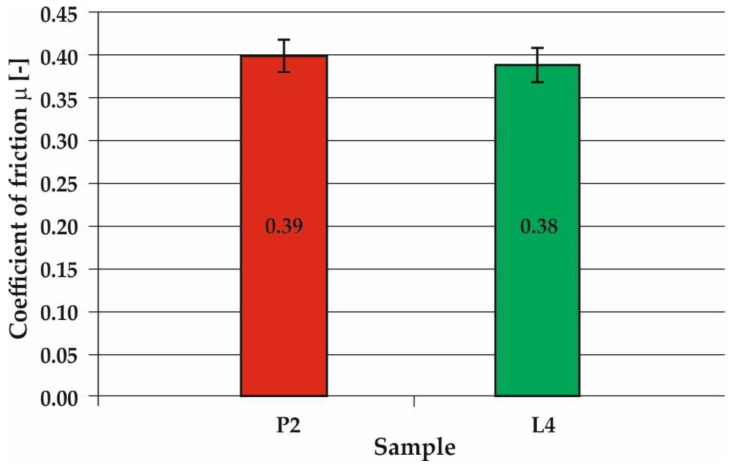
Coefficient of friction for Inconel 625 superalloy layer obtained in the process of plasma powder transferred arc surfacing (sample P2) and of laser surfacing (sample L4).

**Figure 10:**
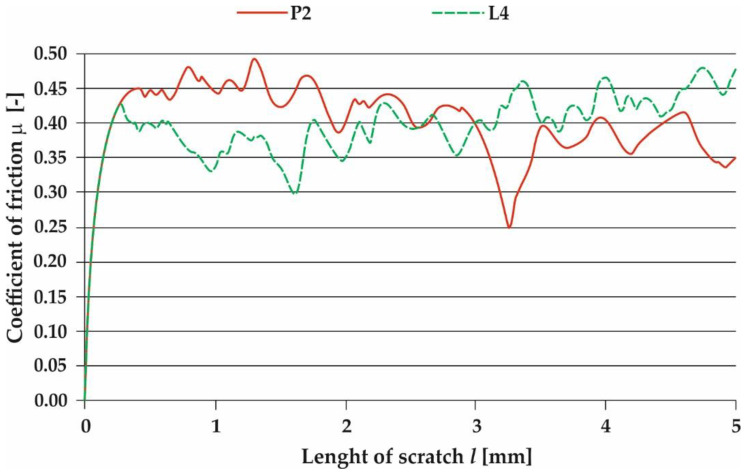
Coefficient of friction as a function of scratch length for Inconel 625 superalloy layers obtained in the process of plasma powder transferred arc surfacing (sample P2) and of laser surfacing (sample L4).

Scratch tests enabled the determination of kinetic friction coefficient *µ*, scratch depth *h*, and friction force T on the outer surface of the surfaced layer. For scratch testing, one sample from powder plasma surfaced and powder laser surfaced pipes were chosen. The surfaced layers subjected to scratch testing had a comparable iron content of under 7%. Scratch test was performed on slightly leveled by grinding surface to exclude the impact of layers convexity on the results. As a result, powder laser surfaced layer (sample L4), with mean iron weight content of 5.1%, has slightly lower (0.38) mean outer surface kinetic coefficient of friction, than powder plasma surfaced layer (sample P2, Fe—5.2 wg.%), for which mean outer surface kinetic coefficient of friction was 0.39. Obtained results correlate with a higher mean microhardness of the sample L4 surfaced layer (Table 10 and Table 11). In cases of both P2 and L4 scratch test results regions with a lower and higher coefficient of friction are visible. These results can be attributed to changes in surface scratch resistance due to partial heat treating of the previous surfacing pass by the next pass. The scratch test results are presented in Figure 9 and Figure 10.

## 4. Conclusions

The aim of this study was to compare robotized powder plasma transferred arc surfacing and powder high power diode laser surfacing parameters on structure, chemical composition, geometry, base material content and mechanical properties of nickel-based superalloy Inconel 625 layers surfaced on 16Mo3 pressure vessel grade steel pipe. The carried comparative analysis enabled the formation of the following conclusions:

1. In the case of both powder plasma surfacing and powder laser surfacing narrow parameter range enabling the formation of nickel-based superalloy Inconel 625 layer with dependable fusion into 16Mo3 steel pipe base material, minimal content of base material, iron content on the outer surfaced layer surface under 7 wg.%, and low HAZ depth is present.
2. Achieving low base material content in the surfaced layer and reducing detrimental microstructural changes in the base material is possible, even when high surfacing linear energy is used, by application of intensive liquid cooling of inner pipe surface during the process.
3. Singular layer depth in case of powder plasma and laser surfacing was two times lower than in the case of MIG surfacing [2,10], moreover finishing machining overmeasure can be reduced in the case of PPTA and HPDDL surfacing. As a result, additional material usage can be reduced in the case of tested technologies.
4. In the powder plasma surfaced and powder laser surfaced layers no additional phases, aside from Ni-Si, with lattice parameters close to pure Nickel were found.
5. The nickel-based superalloy Inconel 625 surfaced layers manufactured by powder laser surfacing exhibit higher hardness compared to powder plasma surfaced layers.

In the future, results of holding temperature impact on nickel-based superalloy Inconel 625 layer will be presented.

## Figures and Tables

**Table 1:** Chemical composition of 16Mo3 steel according to manufacturer data (Margo Ltd., Stalowa Wola, Poland).

Chemical Composition, wg.%											
C	Si	Mn	P	S	N	Cr	Cu	Mo	Ni	Fe	CE ^1)^
0.12–0.2	≤0.35	0.4–0.9	≤0.025	≤0.01	≤0.012	≤0.3	≤0.3	0.25–0.35	≤0.3	Bal.	0.52

Notes: ^1)^ carbon equivalent calculated according to International Institute of Surfacing (IIW) guidelines.

**Table 2:** Chemical composition of 16Mo3 steel according to spectrometric analysis.

Chemical Composition, wg.%										
C	Si	Mn	P	S	N	Cr	Cu	Mo	Ni	Fe
0.17	0.34	0.58	0.022	0.008	0.01	0.26	0.19	0.32	0.28	Bal.

Notes: In the table mean values of 5 measurements were presented.

**Table 3:** Chemical composition of Inconel 625 superalloy; Böhler L625 (EN NiCr22Mo9Nb) powder according to manufacturer data (voestalpine Böhler Surfacing Germany GmbH, Hamm, Germany).

Chemical Composition, wg.%												
C	Si	Mn	P	S	Cr	Mo	Co	Ti	Al	Nb+Ta	Fe	Ni
≤0.03	≤0.4	≤0.5	≤0.01	≤0.01	21–23	8–10	≤1	≤0.4	≤0.4	3.2–3.8	≤5	Bal.

**Table 4:** Chemical composition of Inconel 625 superalloy, Böhler L625 (EN NiCr22Mo9Nb) powder deposit weld according to spectrometric analysis.

Chemical Composition, wg.%												
C	Si	Mn	P	S	Cr	Mo	Ni	Co	Ti	Al	Nb+Ta ^1)^	Fe
0.03	0.26	0.38	0.006	0.004	20.98	8.46	63.37	0.74	0.32	0.17	3.42	1.86

Notes: In the table mean values of 5 measurements were presented, ^1)^ no Tantalum was identified.

**Table 5:** Mechanical properties of Böhler L625 (EN NiCr22Mo9Nb) powder deposit weld.

Mechanical Properties of Deposit Weld ^1)^							
Hardness, HB 30	Yield Strength *R_p_*_0,2_, MPa	Ultimate Tensile Strength *R_m_*, MPa	Elongation *A*_5_, %	Maximum Operation Temperature *T*, °C	Young Modulus *E*, GPa	Impact Energy ISO-V *KV*, J	
210 (≤240)	540 (≥460)	800 (≥760)	38 (≥35)	1000	200 (≤209)	+20 °C	–196 °C
						160	130 (≥32)

Notes: ^1)^ metal in as welded condition (without PWHT), test temperature 20 °C, unless otherwise specified.

**Table 6:** Processing parameters of robotic plasma powder transferred arc surfacing of Inconel 625 superalloy layer on the outer surface of the 16Mo3 steel pipe.

Parameters	Sample Designation								
	P1	P2	P3	P4	P5	P6	P7	P8	P9
Surfacing current *I*, (A)	160	160	160	170	170	170	190	190	190
Arc voltage *U*, (V)	20	20	20	20	20	20	20	20	20
Surfacing velocity *v* ^1)^, (mm/s)	5.2	6.5	7.9	7.9	7.9	9.2	11.8	14.4	14.4
Powder feed rate *q*, (g/min)	17	19	17	15	21	21	21	25	27
Plasma gas flow rate *Q_p_* ^2)^, (l/min)	1.5	1.5	1.5	1.5	1.5	1.5	1.5	1.5	1.5
Shielding gas flow rate *Q_o_* ^2)^, (l/min)	12	12	12	12	12	12	12	12	12
Transport gas flow rate *Q_s_* ^2)^, (l/min)	4	4	4	4	4	4	4	4	4
Nozzle-workpiece distance *l*, (mm)	5	5	5	5	5	5	5	5	5
Overlap ratio, (%)	33	33	33	33	33	33	33	33	33
Heat input *E_u_* ^3)^, (J/mm)	642	514	428	455	455	390	339	277	277

Notes: ^1)^ defined as the resultant velocity of rotational pipe movement and linear industrial robot manipulator movement parallel to pipe rotation axis, ^2)^ Argon 5.0 (99.999%) acc. ISO 14175—I1: 2009 [43] was used as plasma, shielding and transport gas, ^3)^ calculated acc. to the formula: E_u = k∙(U × I)/v The thermal efficiency coefficient for plasma transferred arc k = 0.6 was used.

**Table 7:** Processing parameters of robotic laser powder surfacing of Inconel 625 superalloy layer on the outer surface of the 16Mo3 steel pipe.

Parameters	Oznaczenie Próbki								
	L1	L2	L3	L4	L5	L6	L7	L8	L9
Laser power, (W)	1200	1400	1600	1600	1000	1000	1000	1000	1200
Surfacing velocity ^1)^, (mm/s)	3.9	3.9	3.9	4.7	3.9	3.3	3.3	2.6	2.6
Powder feed rate, (g/min)	10	10	10	15	10	10	7.5	5	5
Shielding gas flow rate *Q_o_* ^2)^, (l/min)	12	12	12	12	12	12	12	12	12
Transport gas flow rate *Q_s_* ^2)^, (l/min)	2	2	2	2	2	2	2	2	2
Overlap ratio, (%)	33	33	33	33	33	33	33	33	33
Heat input ^3)^, (J/mm)	308	359	410	340	256	303	303	385	462

Notes: ^1)^ defined as the resultant velocity of rotational pipe movement and linear industrial robot manipulator movement parallel to pipe rotation axis, ^2)^ Argon 5.0 (99.999%) acc. ISO 14175—I1: 2009 [43] was used as shielding and transport gas, ^3)^ defined as the laser power divided by the traverse speed.

**Table 8:** The average thickness of the surfaced layers, iron content in the coating layers and HAZ width for samples obtained in the process of plasma powder transferred arc surfacing (PPTA), Table 6.

Sample Designation	P1 ^1)^	P2	P3	P4	P5	P6	P7	P8	P9
Average surfacing thickness *g*, (µm)	1275	1339	1269	1132	1416	1193	1292	1392	1646
Average visible HAZ depth *s*, (µm)	1252	719	743	795	831	742	715	633	622
Surfaced layer base metal content ^2)^, *U* (%)	10.7	5.0	3.2	4.2	3.7	5.1	4.1	4,0	3.8
Iron content in surfaced layer ^3)^, *Fe* (wt. %)	11.8	5.2	4.0	4.8	4.2	5.5	4.5	4.3	4.3

Notes: ^1)^ Sample was not considered consecutive examinations as iron content was to high, ^2)^ base metal content in surfaced layer was calculated by formula U = P/(S + P) where P—fused material area on cross-section S—added material area on cross-section P, ^3)^ mean value of five measurements.

**Table 9:** The average thickness of the coating layers, iron content in the coating layers and HAZ width for samples obtained in the process of laser surfacing, Table 7.

Sample Designation	L1 ^1)^	L2 ^1)^	L3 ^1)^	L4	L5	L6	L7 ^1)^	L8 ^1)^	L9 ^1)^
Average surfacing thickness *g*, (µm)	610	810	770	1143	650	1000	180	260	320
Average visible HAZ depth *s*, (µm)	1030	1128	1330	841	1070	1180	1120	1350	1340
Surfaced layer base metal content ^2)^, *U* (%)	11.5	13.6	14.0	3.9	4.8	6.9	12.4	20.6	29.8
Iron content in surfaced layer ^3)^, *Fe* (wt. %)	10.4	14.9	15.1	5.1	6.8	7.5	8.9	19.1	28.7

Notes: ^1)^ Sample was not considered consecutive examinations as iron content was to high, ^2)^ base metal content in surfaced layer was calculated by formula U = P/(S + P) where P—fused material area on cross-section S—added material area on cross-section P, ^3)^ mean value of five measurements.

**Table 10:** Average microhardness HV0.3 measured on the cross-section of 16Mo3 steel pipes plasma clad with Inconel 625 superalloy powder (Table 6).

Microhardness Test Area	Sample Designation								
	P1	P2	P3	P4	P5	P6	P7	P8	P9
	Mean Microhardness HV 0.3								
Base material (16Mo3)	161.5	165.3	163.8	162.7	162.9	163.1	162.9	161.4	160.7
Heat affected zone	209.8	193.4	196.4	199.2	190.5	186.1	182.2	180.3	178.8
Surfaced layer (Inconel 625)	242.8	245.4	241.0	246.2	248.7	249.6	242.2	245.1	242.9

**Table 11:** Average microhardness HV0.3 measured on the cross-section of 16Mo3 steel pipes laser clad with Inconel 625 superalloy powder (Table 7).

Microhardness Test Area	Sample Designation								
	L1	L2	L3	L4	L5	L6	L7	L8	L9
	Mean Microhardness HV0.3								
Base material (16Mo3)	163.5	166.3	168.2	161.8	160.3	164.0	163.7	160.6	163.2
Heat affected zone	259.5	249.7	240.5	251.2	253.7	253.0	250.5	250.7	249.9
Surfaced layer	234.2	231.4	229.0	265.9	254.3	252.0	245.2	204.1	186.7
